# Six-month outcomes and effect of pulmonary rehabilitation among patients hospitalized with COVID-19: a retrospective cohort study

**DOI:** 10.1080/07853890.2021.2001043

**Published:** 2021-11-12

**Authors:** Yaoshan Dun, Chao Liu, Jeffrey W. Ripley-Gonzalez, Ping Liu, Nanjiang Zhou, Xun Gong, Baiyang You, Yang Du, Jiyang Liu, Bo Li, Suixin Liu

**Affiliations:** a Division of Cardiac Rehabilitation, Department of Physical Medicine & Rehabilitation, Xiangya Hospital of Central South University, Changsha, China; b National Clinical Research Centre for Geriatric Disorders, Xiangya Hospital of Central South University, Changsha, China; c Division of Preventive Cardiology, Department of Cardiovascular Medicine, Mayo Clinic, Rochester, MN, USA; d The First Hospital of Changsha, Changsha, China; e Department of Neurology, Xiangya Hospital of Central South University, Changsha, China

**Keywords:** COVID-19, rehabilitation, exercise, immunity, biomarker, respiration

## Abstract

**Background:**

Patients appear to maintain sequelae post-coronavirus disease 2019 (COVID-19) affecting daily life and physical health. We investigated the changes in and the effects of pulmonary rehabilitation (PR) on exercise capacity and immunology six months after COVID-19 hospitalization.

**Methods:**

This retrospective cohort reviewed 233 COVID-19 patients admitted from 17 January 2020 to 29 February 2020. Ninety-eight patients who completed 2-week and 6-month follow-ups and tests were included. Among 98 patients, 27 completed at least five sessions of PR at the First Hospital of Changsha, China, during the 6-month convalescence were allocated to the PR group; the reminder who had not performed any PR were assigned to the control group. The primary outcome was the change in six-minute walk distance (6-MWD) between the 2-week and 6-month follow-ups, which was assessed via analysis of covariance with a covariate of propensity score that adjusted for the potential confounders. Secondary outcomes were the changes in 6-MWD, SARS-CoV-2 immunoglobulins, T-lymphocytes and blood chemistry, which were evaluated via paired tests.

**Results:**

Participants’ ages ranged from 19 to 84 years (*M* = 47, standard deviation (SD)=15) 45.9% identified as male. During the 6-month convalescence, 6-MWD increased 27.0%, with a mean [95% CI] of 113 [92–134] m (*p <* .001). SARS-CoV-2 IgG and IgM decreased 33.3% (*p* = .002) and 43.8% (*p* = .009), CD4+ T cells increased 7.9% (*p* = .04), and the majority of blood chemistry significantly changed. The patients in the PR group acquired a greater increase in 6-MWD than those in control (unadjusted, 194 [167–221] m, *p* < .001; adjusted, 123 [68–181] m, *p* < .001), dose-responsiveness of PR on 6-MWD was observed (*p <* .001). No differences in immunity variables and blood chemistry were observed between groups.

**Conclusions:**

These findings suggest PR may be a strategy to promote the improvement of exercise capacity after COVID-19.

## Introduction

The coronavirus disease 2019 (COVID-19) has infected over 200 million people worldwide as of October 2021. From the patients infected, a proportion, in the convalescence period, appear to maintain multiple sequelae, including symptoms of the virus and related health problems [1–3].

Persisting limitations in respiratory function and gas exchange are pronounced in COVID-19 survivors [1]. Case reports, short-term studies and reviews have shown that COVID-19 shares characteristics with other diseases, including pneumonia, resulted from the coronavirus-1 (SARS-CoV-1) and acute respiratory distress syndrome in that for a time after the infections, patients may present impaired pulmonary function and exercise capacity [3,4], and reinfection from the virus [5]. Our previous study revealed that the content of SARS-CoV-2 specific immunoglobulins significantly decreased in COVID-19 patients 3-month after discharge [6]. Data on longer-term recovery from COVID-19 is limited. An ongoing UK-based study and a study by Huang et al. [1] found that some COVID-19 survivors were troubled by continual muscle fatigue or weakness, sleep difficulties, and anxiety or depression six months after acute infection comparable to severe respiratory syndromes [7].

Therefore, a comprehensive multidisciplinary program is needed to target these conditions. Pulmonary rehabilitation (PR) is a comprehensive intervention intended to improve an individual’s physical and psychological conditions through tailored therapies which include, but are not limited to exercise training, behaviour change and education. The efficacy of PR in promoting recovery in patients with pneumonia other than COVID-19 has been evidenced [8,9]. Therefore, organizations and researchers have called for PR post-COVID-19 infection as a much-needed measure [10,11].

Presently, few studies have reported PR in COVID-19 patients, and interventions are primarily limited to home-based telecommunication interventions [12] and focus on short-term function outcomes, such as respiratory function, mental health status and quality of life [12,13]. However, the effectiveness of PR is in bringing about improvements in physiological outcomes such as exercise capacity, the changes in immunity and blood chemistry over more extended periods remain uncertain.

Therefore, this retrospective cohort study aims to demonstrate the changes in exercise capacity, SARS-CoV-2 specific immunoglobulins, T lymphocytes and blood chemistry and to explore PR’s effects on these outcomes in COVID-19 patients six months after hospital discharge.

## Materials and methods

### Study design and participants

**Figure 1.**
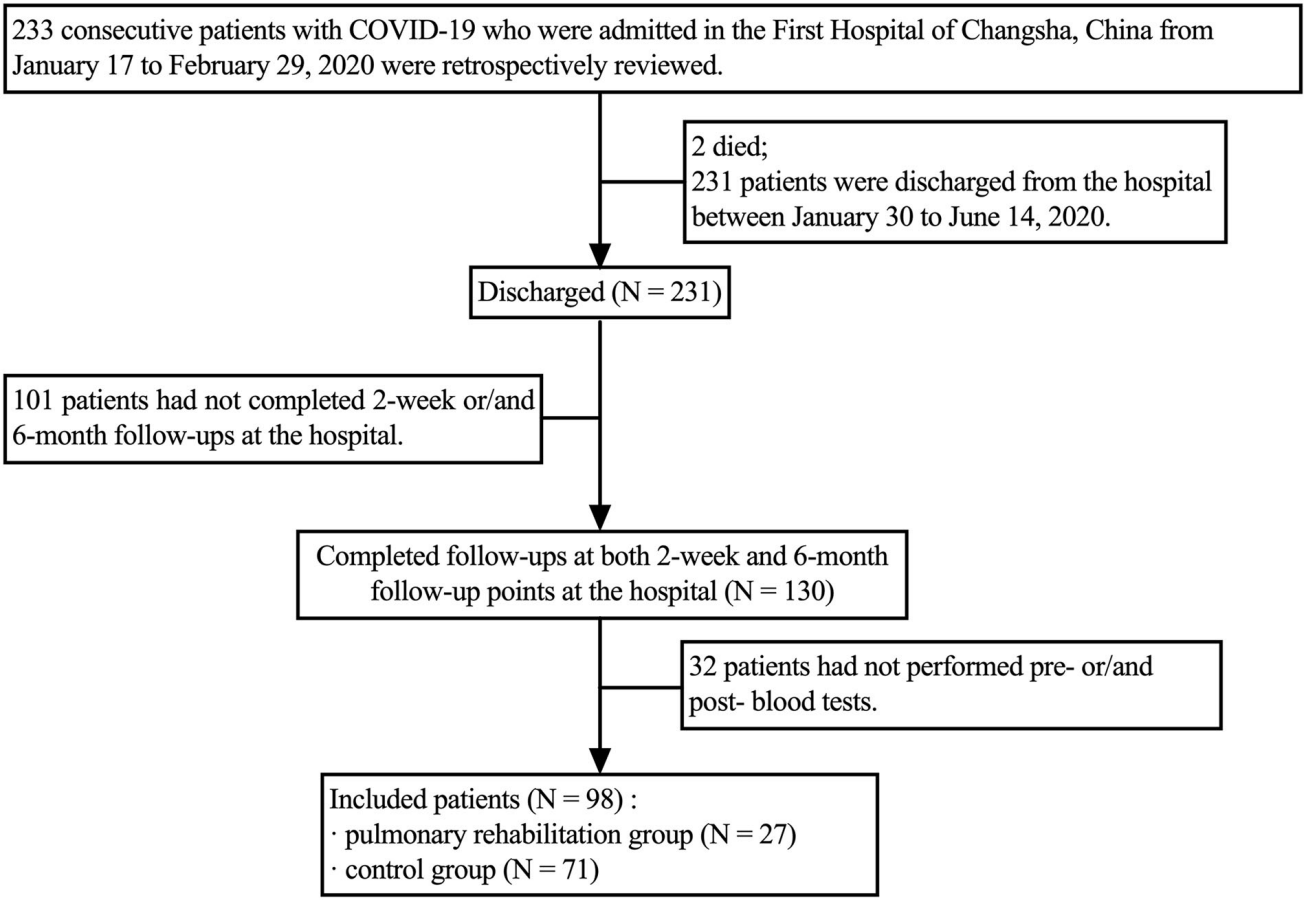
Flowchart of participants. COVID-19: coronavirus disease 2019; SARS-CoV-2: severe acute respiratory syndrome coronavirus 2.

We reviewed the charts of 233 consecutive patients with COVID-19 admitted to the First Hospital of Changsha, China, from 17 January 2020 to 29 February 2020. The average length of stay in the hospital was 18 ± 9 days. Two patients died during hospitalization, 231 were discharged. The criteria for COVID-19’s diagnosis, clinical classification and discharge have been described previously [6]. Inclusion criteria: (i) discharged COVID-19 patients aged ≥18; (ii) volunteered and signed informed consent; (iii) completed 2-week and 6-month follow-ups and performed blood tests. We excluded: (i) patients with cognitive impairment that may result in an inability to cooperate in PR sessions, assessed by the mini-mental state examination-second edition [14]; (ii) signs of respiratory distress or cardiovascular instability. A total of 98 patients were included for analysis. Those who performed at least five PR sessions during the convalescence were allocated to the PR group (*N* = 27); the remainder who had not completed any PR were assigned to the control group (*N* = 71). A participant flowchart is provided in Figure 1. All patients signed informed consent. This study complies with the Declaration of Helsinki, was approved by the Ethics Committee of the First Hospital of Changsha (approval number KX-2020047), and registered at ClinicalTrials.gov (registration number ChiCTR2000038943).

### Sample size and power

The Power Analysis & Sample Size software, version 15.0 (NCSS, LLC, Kaysville, UT) was used to calculate power based on our sample and the guidelines for interpreting the six-minute walk test (6-MWT) [15] that suggests an improvement of more than 70 m in the six-minute walk distance (6-MWD) is necessary to be 95% confident of significant improvement [16]. Group sample sizes of 71 (control group) and 27 (PR group) achieve 99.2% power to reject the null hypothesis of equal means when the population mean difference is 70 m with a standard deviation (SD) for both groups of 70 and a significance level (alpha) of 0.05 using a two-sided two-sample equal-variance *t*-test.

### Pulmonary rehabilitation

The PR program was provided at no cost for COVID-19 patients, who were recommended to conduct three supervised sessions per week at the rehabilitation centre of The First Hospital of Changsha for 12 consecutive weeks, commencing two weeks after discharge from acute care. Each session included: (i) two sets of inspiratory muscle training; (ii) 30 sets of pursed-lip breathing and active cycle of breathing technique (ACBT); (iii) one set of 30 repetitions of maximal voluntary diaphragmatic contractions in the supine position, placing a medium weight (1–3 kg) on the anterior abdominal wall to resist diaphragmatic descent; iv. two 4-minute high-intensity interval training via bicycle or treadmill interspersed with 4-minute low-intensity interval described previously [17,18].

Inspiratory muscle training was performed using Powerbreathe^®^K5 devices (Powerbreathe International Ltd., Southam, UK) with resistance adapted to the level of the inspiratory muscles trained as described previously [19,20]. Initial training load was set at 50% of strength-index, a reliable measure of respiratory muscle strength [19,20]. The patients were instructed to perform two sets of 30 inspiratory labours, a one-minute gap between sets. Individual bacteria/virus filters were used to avoid cross-infection.

Pursed-lip breathing [21] and ACBT [22] were performed as previously described. Before the first set, each patient had a refresher set with the physiotherapist to optimize the technique. Pursed-lip breathing: (i) relaxed neck and shoulder muscles; (ii) breathe in slowly through the nose for two counts, keeping the mouth closed; (iii) pucker or “purse” lips as if going to whistle; (iv) breathe out slowly and gently through pursed lips while counting to four. ACBT: breathing control, thoracic expansion exercises and huffing.

### Exercise prescription

All patients discharged from acute care were given a post-discharge home-based exercise prescription. Frequency: 3–5 days per week; intensity: 40–80% of predicted maximal heart rate (220 – age bpm) or a 13–16 rating of perceived exertion (RPE, Borg’s scale) [23]; time: 30–50 min per day; recommended exercises: brisk walking, jogging and cycling; volume: 150 min per week; in the first-month post-discharge, patients were recommended intensity of 40–60% predicted maximal heart rate or a 13–14 RPE, then gradually increased to the targeted intensity.

### Blood specimen collection and processing

In clinical practice, 10 mL of blood was aseptically collected through venipuncture into three sterile BD vacutainers (Becton, Dickinson and Company, Franklin Lakes, NJ). Of them, 8 mL of blood in two general vacutainers (non-anticoagulated) was centrifuged, collecting serum for detections of SARS-CoV-2 specific immunoglobulins and blood chemistry, the remaining 2 mL of blood in an anticoagulated vacutainer (ethylenediaminetetraacetic acid, EDTA) was arranged for T lymphocytes detection. Whole blood samples and processed serum were stored at room temperature (20–25 °C). Within six hours after the blood specimen collection, a minimum of 100 μL of serum or whole blood tests were extracted for immunoglobulins, T lymphocytes and biomarkers detections.

### Computed tomography (CT) scan and interpretation

Patients performed a CT scan at the 2-week follow-up time point. The procedures of the CT scan and the interpretation have been described previously [24]. Each lung was divided into upper and lower zones according to the oblique fissure of the lung, and each zone was evaluated in terms of involvement. The degree of involvement within each lung zone was assigned a score ranging from 0 (none) to 100% (complete involvement) of one zone. The parenchymal abnormality on CT images was graded on a four-point scale: 1, normal attenuation; 2, ground-glass attenuation; 3, consolidation; and 4, honeycombing opacity. The ground-glass opacity was defined as an area of hazy increased lung opacity, within which margins of pulmonary vessels may be indistinct. Consolidation appeared as a homogeneous increase in pulmonary parenchymal attenuation that obscured vessels and airway walls’ margins. Honeycombing opacity refers to damaged and fibrotic lung tissue containing numerous cystic airspaces with thick fibrous walls. Points from all zones were added to arrive at a final total cumulative score (range of possible scores 0–16).

### Six-minute walk test

The 6-MWT is a tool for the objective assessment of functional exercise capacity and management in patients with pulmonary diseases [25]. The test has shown good reliability and validity in PR along corridors with the length of 30-m and above [26]. Physiotherapists conducted tests according to the standard procedure [15,25]. Patients were asked to walk along a 45-m trafficked corridor for 6 min, with the primary outcome measure being the 6-MWD measured in metres. Measurements of heart rate, pulse oximetry were also monitored using Welch Allyn Vital Signs Monitor (model 408, Welch Allyn Protocol Inc., Beaverton, OR). Patients were given feedback and encouragement at the end of every minute. Patients were asked to avoid heavy meals two hours before testing, and caffeine, alcohol, tea and intense physical exercise 24 h before.

### Physical activity

Self-reported time spent walking and doing light/moderate/vigorous exercise across various domains, including those prescribed upon hospital discharge, was assessed at the six-month follow-up point, using the seven-item short form of The International Physical Activity Questionnaire (IPAQ-SF) [27]. The IPAQ has demonstrated good reliability with intraclass correlation coefficients ranging from 0.81 to 0.89 in Chinese adults [28]. Total physical activity measured by IPAQ was moderately correlated with pedometer measured steps [28].

### Data collection

Patients’ demographics, clinical characteristics, PR sessions, 6-MWD, values of blood chemistry, SARS-CoV-2 specific immunoglobulins and the number of T lymphocytes, nucleic acid test results of SARS-CoV-2 before, 2-week, and 6-month after discharge were extracted from the electronic medical record system or through asking patients. IPAQ-SF during convalescence was collected from documented files. To protect confidentiality, all patients were codified and anonymized by omitting direct identifiers, such as names and email addresses, and the removal of quasi-identifiers, such as socio-economic information.

### Statistical analysis

The primary outcome was the effect of PR on the change in 6-MWD of COVID-19 patients during 6-month convalescence. The secondary outcomes were the changes in 6-MWD, SARS-CoV-2-specific IgG and IgM, T lymphocytes, and blood chemistry six months post-hospitalization, which were assessed by paired *t*-test and Wilcoxon’s sign rank test accordingly. To evaluate for obvious selection bias resulting from the loss of follow-up, demographics and clinical characteristics of those included and excluded in the study were assessed via independent *t*-test or Chi-square test accordingly.

The difference between the changes in variables during 6-month convalescence across the control and PR groups was initially evaluated using an independent *t*-test or Wilcoxon’s rank-sum test, then further assessed by analysis of covariance (ANCOVA) with a covariate of propensity score [29,30] that adjusted for variables that had a difference between groups with a *p* value <.1, and that may influence results. Sensitivity analysis was performed for age, sex, COVID-19 classification, comorbidity, baseline exercise capacity and physical activity volume per week during 6-month convalescence. To investigate any dose-responsiveness of PR on outcomes, the PR group was divided into two subgroups based on the value of median, low-volume PR (≥5 and <17 sessions) and high-volume PR (>17). Analyses were carried out with the use of SAS software, version 9.4 (SAS Institute, Cary, NC), a two-tailed alpha level of 0.05 was considered significant.

## Results

### Demographics and clinical characteristics of patients

The demographics and clinical characteristics of included 98 patients are summarized in Table 1. The average age was 47 ± 15 years. The patients in the PR group were older than those in the control group (54 ± 16 vs. 44 ± 13 years, mean ± SD, *p* = .002).

**Table 1.** Demographics and clinical characteristics of patients.

	Total (*N* = 98)	Control (*N* = 71)	PR (*N* = 27)	*p* Value*
Male, no. (%)	45 (45.9)	36 (50.7)	9 (33.3)	.12
Age, years	47 ± 15	44 ± 13	54 ± 16	.002
Body weight, kg	61.2 ± 11.6	65.4 ± 12.6	61.1 ± 7.6	.10
BMI, kg/m^2^	23.8 ± 3.0	23.8 ± 3.2	23.8 ± 2.5	.99
Clinical classification of COVID-19, no. (%)				.08
Mild cases	1 (1.0)	1 (1.4)	0 (0)	
Moderate cases	73 (74.5)	56 (78.9)	17 (63.0)	
Severe cases	18 (18.4)	10 (14.1)	8 (29.6)	
Critical cases	6 (6.1)	4 (5.6)	2 (7.4)	
Length of hospital stay, days	18 ± 9	17 ± 8	18 ± 9	.66
CT scan scores	2 ± 2	2 ± 1	3 ± 1	.11
Smoking status, no. (%)				.56
Smoking	9 (9.2)	7 (9.9)	2 (7.4)	
Previously smoked	1 (1.0)	1 (1.4)	0 (0)	
Never	88 (89.8)	63 (88.7)	25 (92.6)	
Comorbidity, no. (%)^a^	34 (34.7)	21 (29.6)	13 (48.2)	.10
Hypertension	11 (11.2)	8 (11.3)	3 (11.1)	.99
Diabetes mellitus	7 (7.1)	3 (4.2)	4 (14.8)	.09
Dyslipidaemia	8 (8.2)	6 (8.5)	2 (7.4)	.99
Cardiovascular disease	12 (12.2)	6 (8.5)	6 (22.2)	.09
Cerebrovascular disease	3 (3.0)	1 (1.4)	2 (7.4)	.18
Peptic ulcer	3 (3.06)	3 (4.23)	0 (0)	.56
Cancer	4 (4.1)	4 (5.6)	0 (0)	.57
Medications used during hospitalization, no. (%)				
Glucocorticoid	32 (32.7)	20 (28.2)	12 (44.4)	.13
Lopinavir/ritonavir	78 (79.6)	57 (80.3)	21 (77.8)	.78
Arbidol	50 (51.0)	39 (54.9)	11 (40.7)	.21
Interferon	65 (66.3)	50 (70.4)	15 (55.6)	.17
Chloroquine phosphate	18 (18.4)	13 (18.3)	5 (18.5)	.98
Antibiotics	51 (52.0)	33 (46.4)	18 (66.7)	.07
Immunoglobulin	30 (30.6)	21 (29.6)	9 (33.3)	.72
CVD agents^b^	24 (24.5)	15 (21.1)	9 (33.3)	.21
Physical activity, METs·min/week	2484.1 ± 2803.0	2530 ± 2582	2363 ± 3369	.79

PR: pulmonary rehabilitation group; SARS-CoV-2: severe acute respiratory syndrome coronavirus 2; BMI: body mass index; COVID-19: coronavirus disease-19; CT: computed tomography; CVD: cardiovascular disease.

Data are expressed as mean ± standard deviation (SD) for continuous variables and number (%) for categorical variables. Independent *t*-test and Chi-square test were used for assessing the difference between groups in continuous and categorical variables, respectively.

* *p* Value for comparison of control and PR groups.

^a^ Comorbidity accounts for hypertension, diabetes mellitus, dyslipidaemia, cardiovascular disease, cerebrovascular disease, peptic ulcer and cancer.

^b^ CVD agents include anti-platelets, anti-coagulants, beta-blockers, calcium channel blockers, angiotensin-converting enzyme inhibitors/angiotensin II receptor blockers, diuretics, nitrate and digoxin.

**Figure 2.**
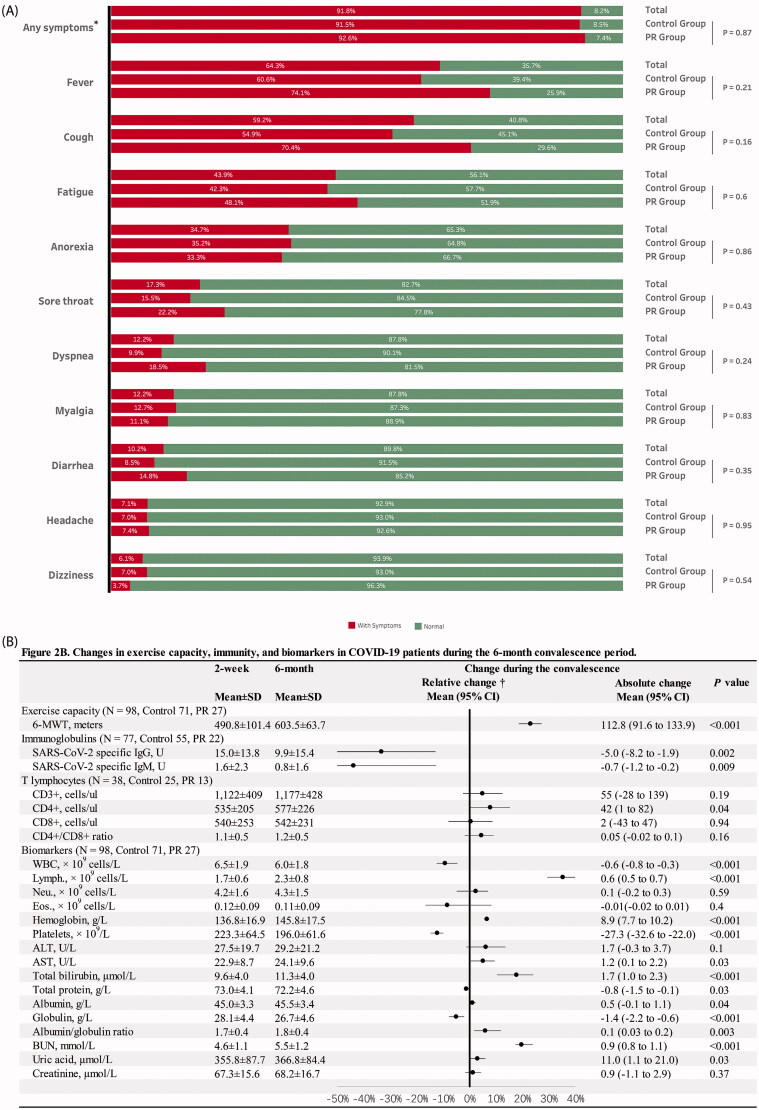
(A) Clinical symptoms in the COVID-19 patients during hospitalization; (B) changes in exercise capacity, severe acute respiratory syndrome coronavirus 2 (SARS-CoV-2) specific immunoglobulins, T lymphocytes and biochemical and haematological biomarkers in the COVID-19 patients during the 6-month convalescence period. COVID-19: coronavirus disease-19; SD: standard deviation; 95% CI: 95% confidence interval; 6-MWD: six-minute walk distance; WBC: white blood cells; CD3+: mature human T lymphocytes; CD4+: helper/inducer T lymphocytes; CD8+: suppressor/cytotoxic T lymphocytes; WBC: white blood cell; Lymph.: lymphocytes; Neu.: neutrophils; Eos.: eosinophils; ALT: alanine aminotransferase; AST: aspartate aminotransferase; BUN: blood urea nitrogen. *Any symptoms indicate if a patient has any of the following symptoms: fever, cough, fatigue, anorexia, sore throat, dyspnoea, myalgia, diarrhoea, headache and dizziness. †The value of relative change was calculated by the equation of (value of change/baseline value)×100. The absolute changes in variables during the 6-month convalescence period were assessed by paired *t*-test and Wilcoxon’s sign-rank test for normal and non-normal distribution variables, respectively.

Of the patients included, 91.8% (*N* = 90) presented at least one clinical symptom during hospitalization; as shown in Figure 2(A), the most common symptoms were fever, fatigue and cough; no significant difference was found between the control and the PR groups (91.5% vs. 92.6%, *p* = .87). During the 6-month convalescence, six patients reported symptoms, three complained of lower back pain, two attributed this to kidney stones, one resulted from sciatica, one reported a sore throat related to pharyngitis and two suffered unexplained mild headaches.

No significant difference between excluded (*N* = 133) and included (*N* = 98) patients was found in demographics and clinical characteristics, indicating less susceptibility to selection bias resulting from the loss of follow-up. More details are displayed in Supplementary Table 1. All SARS-CoV-2 nucleic acid test results were negative; no re-infection was identified.

### Changes in exercise capacity, immunity and blood chemistry in COVID-19 patients during the 6-month convalescence period

Figure 2(B) presents absolute and relative changes in 6-MWD, SARS-CoV-2 specific IgG and IgM, T lymphocytes, and blood chemistry of COVID-19 patients during 6-month convalescence. The 6-MWD increased on average from 491 to 604 m with a mean [95% CI] increase of 113 [92–134] m (*p<* .001). The increase relative to baseline was 27.0% [18.7–27.3%].

SARS-CoV-2 specific IgG and IgM significantly decreased by 33.3% (*p=* .002) and 43.8% (*p* = .009), respectively. There was a mild increase in CD4+ T cells (7.9%, *p* = .04). No significant changes in CD3+ T cell, CD8+, CD4+/CD8+ ratio were observed (*p*> .05 for each variable).

The numbers of white blood cells and platelets moderately decreased by 9.2% (*p* < .001) and 12.2% (*p* < .001), while the number of lymphocytes and content of haemoglobin increased by 35.3% (*p* < .001) and 6.5% (*p* < .001). Detailed results are presented in Table 2.

**Table 2.** The effects of pulmonary rehabilitation on exercise capacity, immunity and blood chemistry in COVID-19 patients during the 6-month convalescence period.

	Control group		PR group		Unadjusted analysis^a^		Adjusted analysis^b^	
	Baseline	Change	Baseline	Change	Diff. between changes		Diff. between changes	
	Mean ± SD	Mean ± SD	Mean ± SD	Mean ± SD	95% CI	*p* Value	95% CI	*p* Value
Exercise capacity (*N* = 98, control 71, PR 27)								
6-MWD, m	538 ± 68	60 ± 58	365.8 ± 59.0	254 ± 67	194 (167–221)	<.001	123 (66–181)	<.001
SARS-CoV-2 specific immunoglobulins (*N* = 77, control 55, PR 22)								
IgG, U	13.9 ± 13.5	–5.1 ± 13.4	17.7 ± 14.4	–4.9 ± 15.6	0.3 (–7.4 to 8.0)	.84	–4.0 (–20.7 to 12.7)	.64
IgM, U	1.5 ± 2.5	–0.6 ± 2.6	1.6 ± 1.7	–1.0 ± 1.6	–0.4 (–1.4 to 0.6)	.44	–0.7 (–3.6 to 2.1)	.60
T lymphocytes (*N* = 38, control 25, PR 13)								
CD3+, cells/µL	1161 ± 404	51 ± 271	1045 ± 424	63 ± 231	12 (–160 to 184)	.89	–82 (–462 to 299)	.67
CD4+, cells/µL	550 ± 200	40 ± 137	507 ± 219	45 ± 96	5 (–72 to 82)	.90	37 (–147 to 220)	.69
CD8+, cells/µL	565 ± 250	–4 ± 135	492 ± 262	12 ± 150	17 (–89 to 119)	.74	–107 (–309 to 94)	.29
CD4+/CD8+ ratio	1.1 ± 0.5	0.03 ± 0.2	1.2 ± 0.6	0.09 ± 0.2	0.05 (–0.09 to 0.2)	.46	0.1 (–0.3 to 0.4)	.69
Blood chemistry (*N* = 98, control 71, PR 27)								
WBC, ×10^9^ cells/L	6.5 ± 1.8	–0.5 ± 1.0	6.6 ± 2.1	–0.7 ± 1.2	–0.2 (–0.7 to 0.3)	.43	–1.0 (–2.1 to 0.2)	.10
Lymph., ×10^9^ cells/L	1.8 ± 0.6	0.6 ± 0.6	1.6 ± 0.7	0.6 ± 0.5	0.01 (–0.2 to 0.2)	.98	–0.3 (–0.9 to 0.3)	.34
Neu., ×10^9^ cells/L	4.2 ± 1.5	0.1 ± 1.0	4.4 ± 1.7	–0.1 ± 1.3	–0.3 (–0.9 to 0.3)	.28	–1.0 (–2.2 to 0.3)	.12
Eos., ×10^9^ cells/L	0.13 ± 0.09	–0.01 ± 0.06	0.10 ± 0.09	0.01 ± 0.05	0.01 (–0.02 to 0.03)	.70	0.01 (–0.06 to 0.07)	.94
Haemoglobin, g/L	139.6 ± 14.5	9.5 ± 6.3	129.7 ± 20.7	7.6 ± 6.5	–1.9 (–4.8 to 1.0)	.19	–1.6 (–8.5 to 5.3)	.64
Platelets, ×10^9^/L	225.9 ± 50.0	–28.6 ± 26.7	216.3 ± 93.3	–23.7 ± 26.0	5.0 (–6.9 to 16.9)	.40	17.1 (–11.9 to 46.1)	.24
ALT, U/L	29.3 ± 21.4	1.4 ± 11.7	22.8 ± 13.1	2.3 ± 3.6	0.9 (–2.0 to 3.9)	.58	2.8 (–8.3 to 14.0)	.62
AST, U/L	23.4 ± 9.5	1.3 ± 6.0	21.7 ± 6.0	0.8 ± 2.7	–0.5 (–2.3 to 1.2)	.54	–1.6 (–4.2 to 7.4)	.58
Total bilirubin, μmol/L	9.4 ± 3.9	1.7 ± 3.4	9.8 ± 4.4	1.5 ± 2.6	–0.3 (–1.6 to 1.0)	.67	–0.2 (–3.7 to 3.4)	.93
Total protein, g/L	73.1 ± 3.7	–1.0 ± 3.8	72.8 ± 5.1	–0.3 ± 2.8	0.6 (–0.8 to 2.0)	.36	1.1 (–2.8 to 5.0)	.58
Albumin, g/L	45.0 ± 3.3	0.6 ± 3.2	44.8 ± 3.4	0.5 ± 2.6	–0.1 (–1.3 to 1.2)	.92	1.3 (–2.0 to 4.6)	.45
Globulin, g/L	28.0 ± 4.1	–1.6 ± 3.7	28.0 ± 5.2	–0.9 ± 4.4	0.7 (–1.3 to 2.6)	.47	–0.3 (–4.5 to 4.0)	.90
Albumin/globulin ratio	1.7 ± 0.4	0.1 ± 0.3	1.7 ± 0.4	0.1 ± 0.4	–0.1 (–0.3 to 0.1)	.39	0.1 (–0.3 to 0.5)	.71
BUN, mmol/L	4.5 ± 1.0	0.9 ± 0.8	4.8 ± 1.2	0.9 ± 0.8	0.04 (–0.3 to 0.4)	.80	0.3 (–0.6 to 1.1)	.53
Uric acid, μmol/L	360.9 ± 84.7	12.9 ± 45.9	342.2 ± 95.6	6.2 ± 59.4	–6.7 (–32.3 to 18.9)	.60	–27.6 (–61.1 to 48.5)	.82
Creatinine, μmol/L	67.3 ± 13.2	0.9 ± 10.4	67.3 ± 21.1	0.9 ± 8.4	0.01 (–4.0 to 4.1)	.99	–1.4 (–12.3 to 9.4)	.80

PR: pulmonary rehabilitation; SD: standard deviation; 95% CI: 95% confidence interval; 6-MWD: six-minute walk distance; SARS-CoV-2: severe acute respiratory syndrome coronavirus 2; WBC: white blood cells; CD3+: mature human T lymphocytes; CD4+: helper/inducer T lymphocytes; CD8+: suppressor/cytotoxic T lymphocytes; WBC: white blood cell; Lymph.: lymphocytes; Neu.: neutrophils; Eos.: eosinophils; ALT: alanine aminotransferase; AST: aspartate aminotransferase; BUN: blood urea nitrogen.

^a^ The difference between the changes across groups was assessed by independent *t*-test and Wilcoxon’s rank sum test for normal and non-normal distribution variables, respectively.

^b^ The difference between the changes across groups was evaluated by analysis of covariance (ANCOVA) with a covariate of propensity score that adjusted for sex, age, body weight, baseline exercise capacity (6-MWD), physical activity per week during 6-month convalescence period, and COVID-19 classification, comorbidity and the use of antibiotics during hospitalization.

### The effects of pulmonary rehabilitation on exercise capacity in COVID-19 patients

**Figure 3.**
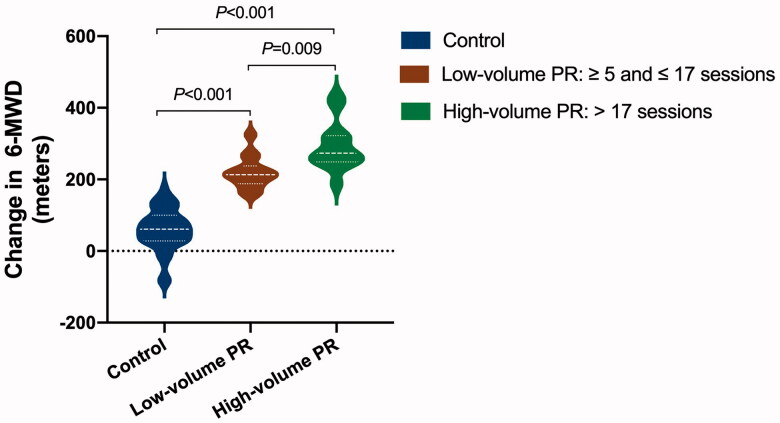
The effect of pulmonary rehabilitation on exercise capacity in COVID-19 patients during the 6-month convalescence period in a dose-dependent way. The difference between changes in 6-minute walk distance (6-MWD) across groups was assessed by analysis of covariance (ANCOVA) with a covariate of propensity score that adjusted for sex, age, body weight, baseline 6-MWD, physical activity per week during the 6-month recovery period, and COVID-19 classification, comorbidity and the use of antibiotics during hospitalization. Post hoc analysis for each comparison was also performed. The mean difference and 95% confidence interval in the high-volume pulmonary rehabilitation group vs. control group are 165 (101–229) m; low-volume vs. control is 88 (22–154); high-volume vs. low-volume is 77 (34–120), *p* values for each assessment are marked on the figure accordingly (*N* = 98, control 71, low-volume PR 14, high-volume PR 13).

Concerning the adherence to PR, the median and interquartile range of the PR sessions completed by 27 patients in the PR group was 17 (10–23). The lowest and highest sessions of PR completed were 5 and 30, respectively. Analyses demonstrated a significantly greater increase in 6-MWD in the PR group than control (unadjusted, 194 [167–221] m, mean difference [95% CI], *p* < .001; adjusted, 123 [68–181]) m, *p* < .001). In COVID-19 severity stratified analysis, patients with moderate COVID-19 in the PR group still showed a greater increase in the 6-MWD than those in the control group (136 [76–196] m, *p* < .001). Due to sample size limitations, the present study had not performed subgroup analyses for patients with mild, severe and critical COVID-19. Dose-responsiveness of PR on 6-MWD (*p* < .001) details is presented in Figure 3.

### The effects of pulmonary rehabilitation on immunity and blood chemistry in COVID-19 patients

No significant differences were found between the PR and control groups in SARS-CoV-2 specific IgG and IgM, CD3+ T cell, CD8+ cell, CD8+ T cell, CD4+/CD8+ ratio and all biomarkers reported in the present study (*p*> .05 for each variable).

## Discussion

This study observed that PR improved exercise capacity in COVID-19 patients in a dose–response fashion. Furthermore, we demonstrated the changes in exercise capacity, SARS-CoV-2 specific immunoglobulins, T lymphocytes and blood chemistry in COVID-19 patients throughout 6-month convalescence.

Exercise capacity is a strong predictor of cardiovascular and all-cause mortality [31]. Impairment of exercise capacity and decreased 6-MWD are likewise associated with greater risks of morbidity and mortality in the general population [32] and patients with various lung diseases [25]. A concerning amount of COVID-19 survivors have experienced decreased exercise capacity as a result of irreversible lung damage linked to COVID-19 [33] and prolonged recovery due to factors during convalescence. As reported by Huang et al., 54.3% of 57 severe and non-severe COVID-19 patients had abnormal CT findings, and 75.4% had abnormal pulmonary function tests following discharge [34]. Studies have suggested that the lung’s ability to transport oxygen to the blood could be significantly compromised post-hospitalization, whereby COVID-19 patients may have residual fibrotic lesions in the pulmonary system subsequent to treatment and hospital discharge, limiting respiratory function [33]. Follow-up research on 143 patients has reported that only 12.6% of their patient population were free from symptoms, and a large percentage experienced dyspnoea, dry cough and other symptoms months following discharge [35]. Another study demonstrated that up to 63% of patients exhibited symptoms of fatigue six months post-hospitalization, 26% remained with sleep difficulties [1]. Furthermore, during treatment, patients in the intensive care unit remain bedridden for extensive periods, with little muscular activation, which could lead to muscle weakness, myopathy, dysphagia with consequent reductions in free-living physical activities and exercise capacity [36].

As a result, health professionals and bodies have expressed the need for PR in patients facing complications in recovery [10,11, 37]. Comprehensive PR [38], including its fundamental components [39]: respiratory muscle training, breathing techniques and exercise training, have been evidenced in improving long-term health outcomes, such as mortality, quality of life, lung function, exercise capacity, in patients with lung diseases [40–43]. A 6–8 week program of PR, emphasizing respiratory training, has been shown to improve 6-MWD in general patients, as reported by Giansanti and Maccioni [44]. More recently, in a short-term study, Liu et al. [13] reported similar findings in COVID-19 patients, 6-weeks of respiratory training consisting of cough, respiratory muscle, diaphragmatic training, stretching and home exercise improved quality of life and 6-MWD. Our results showed that 6-MWD significantly improved in patients after six months, suggesting a gradual recovery in exercise capacity after COVID-19. However, those in the PR group presented significantly greater improvements in exercise capacity than the control. Moreover, the PR volume was associated with improved exercise capacity in a dose–response relationship. The high-volume group demonstrated a significantly greater increase in exercise capacity than the low-volume group.

The data indicates that patients are still presenting residual impairments even at 6-months post-hospitalization, which agrees with previous studies [1]. However, the present study does not have the power to assess whether the patients had recovered completely due to the lack of data of pre-COVID-19 exercise capacity prior to the COVID-19 pandemic. Further studies, aiming to explore the predictors or effective interventions of restoring/establishing pre-COVID-19 expected functional status are warranted.

We further examined the immunological changes during 6-month convalescence. On the basis that most acute viral infections result in the development of protective immunity [45] and experiences with the 2003 SARS epidemic, researchers hypothesized that CD4+ T cell and neutralizing antibody responses aid in the removal of the acute infection of SARS-CoV-2, and immunological memory against SARS-CoV-2 infection may come from retained T and B cells. Our previous study found that SARS-CoV-2 specific variables, IgG and IgM decreased in patients with COVID-19 over 3 months [6]. In the present study, SARS-CoV-2 specific IgG and IgM decreased significantly over six months, CD4+ T cells mildly increased.

The severity of COVID-19 during hospitalization may significantly influence recovery trajectory in exercise capacity and immunological response in patients. To avoid bias from the severity of COVID-19, we assessed the difference in changes of the outcomes between groups using ANCOVA with a covariate of propensity score that adjusted the severity of COVID-19. In addition, we performed COVID-19 severity stratified analysis. These results suggest that the efficacy of PR on exercise capacity and immunological response were not significantly associated with the severity of COVID-19 in patients who were included in the present study.

Many biochemical and haematological biomarkers may be associated with the severity and progression of COVID-19. According to recent reports [46], the tracking of these may prove valuable for screening, clinical management/adjustment and prevention of serious complications, as well as the monitoring of recovery. There were no observed differences in the biological properties between the PR group compared to the control as participants appear to be recovering gradually and may not be impacted by PR.

COVID-19 may, directly and indirectly, affect different parameters of well-being, including physical [1], psychological [47] and cognitive health [48]. Other multidisciplinary interventions that include research-based techniques to improve health parameters may be beneficial to patients. To that effect, multidisciplinary approaches have been called upon, incorporating facets of PR and other specialities, including psychiatry [49].

This study has several limitations. First, this study can only suggest the potential associations between PR and outcomes, but not the causal effect of PR. Second, although we have adjusted for multiple potential confounding factors in analyses, and subgroup analyses were performed, we cannot rule out potential bias from self-selection of PR. Third, we cannot rule out potential bias from loss of follow-up, although there were no differences in demographics and clinical characteristics between included patients and those who lost at least one follow-up. Fourth, we only reviewed patients from a single hospital for a limited period, resulting in a small sample size, which may limit the generalizability of the findings. Lastly, due to the retrospective nature of this study, physical activity was only collected at the 6-month follow-up point therefore, we were unable to analyse differences in physical activity coming along with improved health. To address these limitations, further prospective long-term observational studies and randomized control trials with large sample sizes and multiple health outcomes, including other biomarkers, at multiple centres are warranted.

## Conclusions

COVID-19 patients showed a gradual improvement in exercise capacity, a significant decline in SARS-CoV-2 specific IgG and IgM, a mild increase in CD4+ T cells, and diverse changes in several biochemical and haematological biomarkers 6-month after hospital discharge. Furthermore, PR’s 6-month effect on improving exercise capacity was observed in COVID-19 patients in a dose–response fashion, which suggests that PR may be a strategy to promote recovery in patients during/after acute COVID-19.

## Supplementary Material

Supplemental Material

## Data Availability

The data that support the findings of this study are available from the corresponding author, SL, upon reasonable request.
