# Supplementary material for: Six-month outcomes and effect of pulmonary rehabilitation among patients hospitalized with COVID-19: a retrospective cohort study
Source: Ann Med. 2021 Nov 12;53(1):2099–109. doi: 10.1080/07853890.2021.2001043 (PMC8592619; doi:10.1080/07853890.2021.2001043)
Supplement: Supplemental Material [file IANN_A_2001043_SM0594.docx]

Supplementary Appendix

**Supplementary Table 1. Demographics and clinical characteristics of patients who were included and excluded.**

|  | Excluded patients | Included patients | *P* value |
| --- | --- | --- | --- |
|  | (N=133) | (N=98) |  |
| Male, no. (percent) | 74 (55.6) | 45 (45.9) | 0.18 |
| Age, yrs | 47±16 | 47±15 | 0.87 |
| Body weight, kg | 64.4±13.8 | 64.2±11.6 | 0.88 |
| BMI, kg/m^2^ | 23.8±4.5 | 23.75±3.0 | 0.91 |
| Clinical classification of COVID-19, no. (percent) | | | 0.07 |
| Mild cases | 8 (6.0) | 1 (1.0) |  |
| Moderate cases | 101 (75.9) | 73 (74.5) |  |
| Severe cases | 20 (15.0) | 18 (18.4) |  |
| Critical cases | 4 (3.0) | 6 (6.1) |  |
| Length of hospital stay, days | 19±14 | 18±9 | 0.35 |
| Smoking status, no. (percent) |  |  | 0.78 |
| Smoking | 10 (7.5) | 9 (9.2) |  |
| Previously smoked | 2 (1.5) | 1 (1.0) |  |
| Never | 121 (91.0) | 88 (89.8) |  |
| Comorbidity *, no. (percent) | 41 (30.8) | 34 (34.7) | 0.57 |
| Hypertension | 24 (18.1) | 11 (11.2) | 0.19 |
| Diabetes mellitus | 15 (11.4) | 7 (7.1) | 0.37 |
| Dyslipidemia | 4 (3.0) | 8 (8.2) | 0.13 |
| Cardiovascular disease | 25 (18.8) | 12 (12.2) | 0.87 |
| Cerebrovascular disease | 4 (3.0) | 3 (3.0) | 0.99 |
| Peptic ulcer | 2 (1.5) | 3 (3.06) | 0.65 |
| Cancer | 2 (1.5) | 4 (4.1) | 0.41 |
| Medications used during hospitalization, no. (percent) | | | |
| Glucocorticoid | 37 (27.8) | 32 (32.7) | 0.46 |
| Lopinavir/Ritonavir | 105 (78.9) | 78 (79.6) | 0.99 |
| Arbidol | 58 (43.6) | 50 (51.0) | 0.29 |
| Interferon | 100 (71.2) | 65 (66.3) | 0.14 |
| Chloroquine phosphate | 27 (20.5) | 18 (18.4) | 0.74 |
| Antibiotics | 73 (54.9) | 51 (52.0) | 0.69 |
| Immunoglobulin | 40 (30.1) | 30 (30.6) | 0.99 |
| CVD agents † | 25 (18.8) | 24 (24.5) | 0.33 |

SARS-CoV-2, severe acute respiratory syndrome coronavirus 2; BMI, body mass index; COVID-19, coronavirus disease-19; CVD, cardiovascular disease. Data are expressed as mean ± standard deviation (SD) for continuous variables and number (percent) for categorical variables. Independent *t*-test and Chi-square test were used for assessing the difference between groups in continuous and categorical variables, respectively.

* Comorbidity accounts for hypertension, diabetes mellitus, dyslipidemia, cardiovascular disease, cerebrovascular disease, peptic ulcer and cancer.

† CVD agents include anti-platelets, anti-coagulants, Beta-blockers, calcium channel blockers, angiotensin-converting enzyme inhibitors/angiotensin II receptor blockers, diuretics, nitrate, Digoxin.
